# Supplementary material for: Comparative genomic, transcriptomic, and proteomic reannotation of human herpesvirus 6
Source: BMC Genomics. 2018 Mar 20;19:204. doi: 10.1186/s12864-018-4604-2 (PMC5859498; doi:10.1186/s12864-018-4604-2)
Supplement: Supplementary file 1 — Table S1. List of samples sequenced in this study and associated accession numbers. (DOCX 72 kb) [file 12864_2018_4604_MOESM1_ESM.docx]

Japan

| HSCT patients | |  |  |  | | | | |  |  | |  |  |
| --- | --- | --- | --- | --- | --- | --- | --- | --- | --- | --- | --- | --- | --- |
| ID | sampling date | Genbank accession # | | | |  |  |  |  |  |  |  |  |
| B1 | 2015-Dec | KY274496 | | | |  |  |  |  |  |  |  |  |
| B2 | 2015-Nov | KY274497 | | | |  |  |  |  |  |  |  |  |
| B3 | 2015-Nov | KY274498 | | | |  |  |  |  |  |  |  |  |
| B4 | 2015-Nov | KY274499 | | | |  |  |  |  |  |  |  |  |
| B5 | 2015-Nov | KY274500 | | | |  |  |  |  |  |  |  |  |
| B7 | 2015-Nov | KY274501 | | | |  |  |  |  |  |  |  |  |
| B8 | 2015-Oct | KY274502 | | | |  |  |  |  |  |  |  |  |
| B9 | 2015-Aug | KY274503 | | | |  |  |  |  |  |  |  |  |
| B10 | 2015-July | KY274504 | | | |  |  |  |  |  |  |  |  |
| B11 | 2015-July | KY274505 | | | |  |  |  |  |  |  |  |  |
|  |  |  |  |  | | | | |  |  | |  |  |
|  | | | | | | | | | | | | |  |
|  |  |  |  |  | | | | |  |  | |  |  |
|  |  |  |  |  | | | | |  |  | |  |  |
| ES patients |  |  |  |  | | | | |  |  | |  |  |
| ID | sampling date | Genbank accession # | | | | |  |  | | |  |  |  |
| A11 | 2015-Oct | KY274495 | | |  | |  |  | | |  |  |  |
| A10 | 2015-Nov | KY274494 | | |  | |  |  | | |  |  |  |
| A9 | 2015-Nov | KY274493 | | |  | |  |  | | |  |  |  |
| A8 | 2015-Nov | KY274492 | | |  | |  |  | | |  |  |  |
| A7 | 2015-Nov | KY274491 | | |  | |  |  | | |  |  |  |
| A5 | 2015-Nov | KY274490 | | |  | |  |  | | |  |  |  |
| A4 | 2015-Dec | KY274489 | | |  | |  |  | | |  |  |  |
| A3 | 2015-Dec | KY274488 | | |  | |  |  | | |  |  |  |
| A2 | 2015-Dec | KY274487 | | |  | |  |  | | |  |  |  |
| A1 | 2015-Dec | KY239023 | | |  | |  |  | | |  |  |  |

New York

| **ID** | **Number of Passes** | **Race / Ethnicity** | | | **Location** | **Genbank accession #** |  |  |  |  |  |  |  |  |  |  |  |  |  |
| --- | --- | --- | --- | --- | --- | --- | --- | --- | --- | --- | --- | --- | --- | --- | --- | --- | --- | --- | --- |
| 32 | 3 | White | | | NY | KY290187 |  |  |  |  |  |  |  |  |  |  |  |  |  |
| 40 | 2 | White | | | NY | KY290188 |  |  |  |  |  |  |  |  |  |  |  |  |  |
| 104 | 3 | Unknown | | | NY | KY290189 |  |  |  |  |  |  |  |  |  |  |  |  |  |
| 129 | 3 | Unknown | | | NY | KY290190 |  |  |  |  |  |  |  |  |  |  |  |  |  |
| 144 | 3 | Black | | | NY | KY290191 |  |  |  |  |  |  |  |  |  |  |  |  |  |
| 156 | 3 | Black | | | NY | KY290192 |  |  |  |  |  |  |  |  |  |  |  |  |  |
| 200 | 3 | Unknown | | | NY | KY290193 |  |  |  |  |  |  |  |  |  |  |  |  |  |
| 214 | 3 | Unknown | | | NY | KY290194 |  |  |  |  |  |  |  |  |  |  |  |  |  |
| 233 | 4 | Hispanic | | | NY | KY290195 |  |  |  |  |  |  |  |  |  |  |  |  |  |
| 236 | 3 | Unknown | | | NY | KY290196 |  |  |  |  |  |  |  |  |  |  |  |  |  |
| 241 | 2 | Unknown | | | NY | KY290197 |  |  |  |  |  |  |  |  |  |  |  |  |  |
| 275 | 2 | Black | | | NY | KY290198 |  |  |  |  |  |  |  |  |  |  |  |  |  |
| 306 | 2 | White | | | NY | KY290199 |  |  |  |  |  |  |  |  |  |  |  |  |  |
| 309 | 2 | White | | | NY | KY290200 |  |  |  |  |  |  |  |  |  |  |  |  |  |
| 310 | 2 | White | | | NY | KY290201 |  |  |  |  |  |  |  |  |  |  |  |  |  |
| 317 | 2 | White | | | NY | KY290202 |  |  |  |  |  |  |  |  |  |  |  |  |  |
| 319 | 2 | White | | | NY | KY290203 |  |  |  |  |  |  |  |  |  |  |  |  |  |
| 332 | 2 | Black | | | NY | KY290204 |  |  |  |  |  |  |  |  |  |  |  |  |  |
| 335 | 2 | White | | | NY | KY290205 |  |  |  |  |  |  |  |  |  |  |  |  |  |
| 338 | 2 | Black | | | NY | KY290206 |  |  |  |  |  |  |  |  |  |  |  |  |  |
| 350 | 2 | White | | | NY | KY290207 |  |  |  |  |  |  |  |  |  |  |  |  |  |
| 351 | 2 | Hispanic | | | NY | KY290208 |  |  |  |  |  |  |  |  |  |  |  |  |  |
| 353 | 2 | Black | | | NY | KY290209 |  |  |  |  |  |  |  |  |  |  |  |  |  |
| 357 | 3 | Black | | | NY | KY290210 |  |  |  |  |  |  |  |  |  |  |  |  |  |
| 373 | 5 | White | | | NY | KY290211 |  |  |  |  |  |  |  |  |  |  |  |  |  |
| 379 | 3 | Asian | | | NY | KY290212 |  |  |  |  |  |  |  |  |  |  |  |  |  |
| 380 | 4 | White | | | NY | KY290213 |  |  |  |  |  |  |  |  |  |  |  |  |  |
| 390 | 5 | Hispanic | | | NY | KY290214 |  |  |  |  |  |  |  |  |  |  |  |  |  |
| 393 | 2 | White | | | NY | KY290215 |  |  |  |  |  |  |  |  |  |  |  |  |  |
| 394 | 7 | Black | | NY | | KY290216 |  |  |  |  |  |  |  |  |  |  |  |  |  |
| 397 | 4 | White | | NY | | KY290217 |  |  |  |  |  |  |  |  |  |  |  |  |  |
| 399 | 3 | White | | NY | | KY290218 |  |  |  |  |  |  |  |  |  |  |  |  |  |
| 405 | 6 | White | | NY | | KY290219 |  |  |  |  |  |  |  |  |  |  |  |  |  |
| 434 | 4 | Hispanic | | NY | | KY290220 |  |  |  |  |  |  |  |  |  |  |  |  |  |
| 436 | 3 | White | | NY | | KY290221 |  |  |  |  |  |  |  |  |  |  |  |  |  |
|  |  |  |  | | | | |  | |  | |  | |  | |  | |  | |
|  | | | | | | | |  |  | |  | |  | |  | |  | |  |

|  |  |  |  |
| --- | --- | --- | --- |

Fred Hutch

| **ID** | **HHV6 type** | **Race** | **Sample location** | **Accession** |  |
| --- | --- | --- | --- | --- | --- |
| DNRC11 | B | Caucasian | USA | KY315520 |  |
| HP100E10 | B | Caucasian | Unknown | KY290184 |  |
| HP104A5 | A | Caucasian | USA | KY290185 |  |
| HP104C1 | B | Caucasian | USA | KY290186 |  |
| HP10A10 | B | Caucasian | USA | KY274506 |  |
| HP10H9 | B | Caucasian | USA | KY274507 |  |
| HP12F5 | B | Caucasian | USA | KY315528 |  |
| HP12G6 | B | Caucasian | USA | KY315529 |  |
| HP12H12 | B | Caucasian | USA | KY315530 |  |
| HP15A11 | A | Caucasian | USA | KY274508 |  |
| HP15H9 | B | Caucasian | USA | KY274509 |  |
| HP17H8 | B | Unknown | USA | KY274510 |  |
| HP19G7 | B | Caucasian | USA | KY274511 |  |
| HP1F1 | B | Caucasian | USA | KY315521 |  |
| HP21F9 | B | Caucasian | USA | KY274512 |  |
| HP23A7 | A | Caucasian | USA | KY315531 |  |
| HP23G12 | B | Caucasian | USA | KY274513 |  |
| HP24D3 | B | Caucasian | USA | KY274514 |  |
| HP26A11 | B | Caucasian | USA | KY274515 |  |
| HP2C10 | B | Caucasian | USA | KY315522 |  |
| HP30A9 | B | Caucasian | USA | KY274516 |  |
| HP30E3 | B | Caucasian | USA | KY274517 |  |
| HP31C9 | B | Caucasian | UK | KY274518 |  |
| HP33A7 | B | Unknown | UK | KY315532 |  |
| HP33G9 | B | Caucasian | USA | KY274519 |  |
| HP34B2 | B | Caucasian | USA | KY274520 |  |
| HP34B3 | B | Caucasian | USA | KY274521 |  |
| HP34D9 | B | Caucasian | USA | KY274522 |  |
| HP36C6 | B | Unknown | Australia | KY315533 |  |
| HP36G11 | B | Caucasian | USA | KY274523 |  |
| HP38H8 | B | Caucasian | USA | KY274524 |  |
| HP40E6 | B | Caucasian | USA | KY315534 |  |
| HP42C9 | B | Caucasian | USA | KY274525 |  |
| HP43E10 | B | Caucasian | USA | KY315535 |  |
| HP44C1 | B | Caucasian | USA | KY290171 |  |
| HP46B12 | B | Black | USA | KY315536 |  |
| HP49H10 | B | Caucasian | USA | KY290172 |  |
| HP4A11 | B | Caucasian | USA | KY315523 |  |
| HP4C10 | B | Caucasian | USA | KY315524 |  |
| HP51D11 | B | Caucasian | USA | KY290173 |  |
| HP54B4 | B | Caucasian | USA | KY290174 |  |
| HP54H11 | B | Unknown | USA | KY290175 |  |
| HP58A9 | B | Caucasian | USA | KY290176 |  |
| HP58B9 | B | Caucasian | USA | KY290177 |  |
| HP5E3 | B | Unknown | USA | KY315525 |  |
| HP61C11 | B | Caucasian | USA | KY290178 |  |
| HP63F10 | B | Caucasian | USA | KY290179 |  |
| HP67A4 | B | Caucasian | USA | KY290180 |  |
| HP69A10 | B | Caucasian | USA | KY290181 |  |
| HP69F10 | B | Unknown | USA | KY290182 |  |
| HP6G12 | B | Caucasian | USA | KY315526 |  |
| HP71B11 | B | Unknown | Germany | KY315538 |  |
| HP71F1 | B | Caucasian | USA | KY315539 |  |
| HP73C5 | A | Caucasian | USA | KY290183 |  |
| HP73F12 | A | Unknown | Germany | KY315540 |  |
| HP79D8 | B | Caucasian | USA | KY315541 |  |
| HP81C2 | B | Caucasian | USA | KY315542 |  |
| HP81D12 | B | Caucasian | USA | KY315543 |  |
| HP83H7 | B | Caucasian | USA | KY315544 |  |
| HP88H3 | B | Caucasian | USA | KY315546 |  |
| HP8H1 | B | Caucasian | USA | KY315527 |  |
| HP91B10 | B | Caucasian | USA | KY315547 |  |
| HP93H4 | B | Caucasian | USA | KY315548 |  |
| HP94B11 | A | Caucasian | USA | KY315549 |  |
| HP95C5 | B | Caucasian | USA | KY315550 |  |
| HP96H2 | B | Caucasian | USA | KY315551 |  |
| JHPT-B12 | B | Caucasian | USA | KY315553 |  |
| JHPT-C12 | B | Caucasian | USA | KY315554 |  |
| JHPT-D12 | A | Caucasian | USA | KY315555 |  |
| JHPT-D6 | B | Caucasian | USA | KY315556 |  |
| JHPT-E5 | B | Caucasian | USA | KY315557 |  |
| JHPT-G1 | A | Caucasian | USA | KY315558 |  |
| HP88D9 | A | Unknown | USA | KY315545 |  |
| HP96H12 | A | Caucasian | USA | KY315552 | |

Fred Hutch U90

| **ID** | **Collection date** | **Accession** |
| --- | --- | --- |
| AH1 | 2015 | MF624263 |
| BF2 | 2014 | MF624253 |
| BG2 | 2014 | MF624255 |
| AB3 | 2014 | MF624257 |
| AH2 | 2014 | MF624260 |
| AC3 | 2015 | MF624258 |
| AG3 | 2015 | MF624259 |
| AD3 | 2015 | MF624256 |
| BD1 | 2015 | MF624254 |
| AH3 | 2015 | MF624262 |
| AE1 | 2015 | MF624261 |
