# Supplementary material for: Comparative genomic, transcriptomic, and proteomic reannotation of human herpesvirus 6
Source: BMC Genomics. 2018 Mar 20;19:204. doi: 10.1186/s12864-018-4604-2 (PMC5859498; doi:10.1186/s12864-018-4604-2)
Supplement: Supplementary file 2 — Figure S1. Resequencing of select iciHHV-6B specimens confirms identical sequences among unrelated patients. Samples from select iciHHV-6B specimens with identical sequences were re-extracted, re-prepared and re-sequenced from original patient material to rule out contamination or a sample specimen switch during the sequencing process. 11/12 of specimens gave identical sequence throughout the unique long region directly from de novo assembly. One specimen (iciHHV-6B-30E3) had one nucleotide change (G77564 T) upon resequencing at a base that had a G/T variant allele frequency of approximately 50% each time the sample was sequenced. (PDF 145 kb) [file 12864_2018_4604_MOESM2_ESM.pdf]

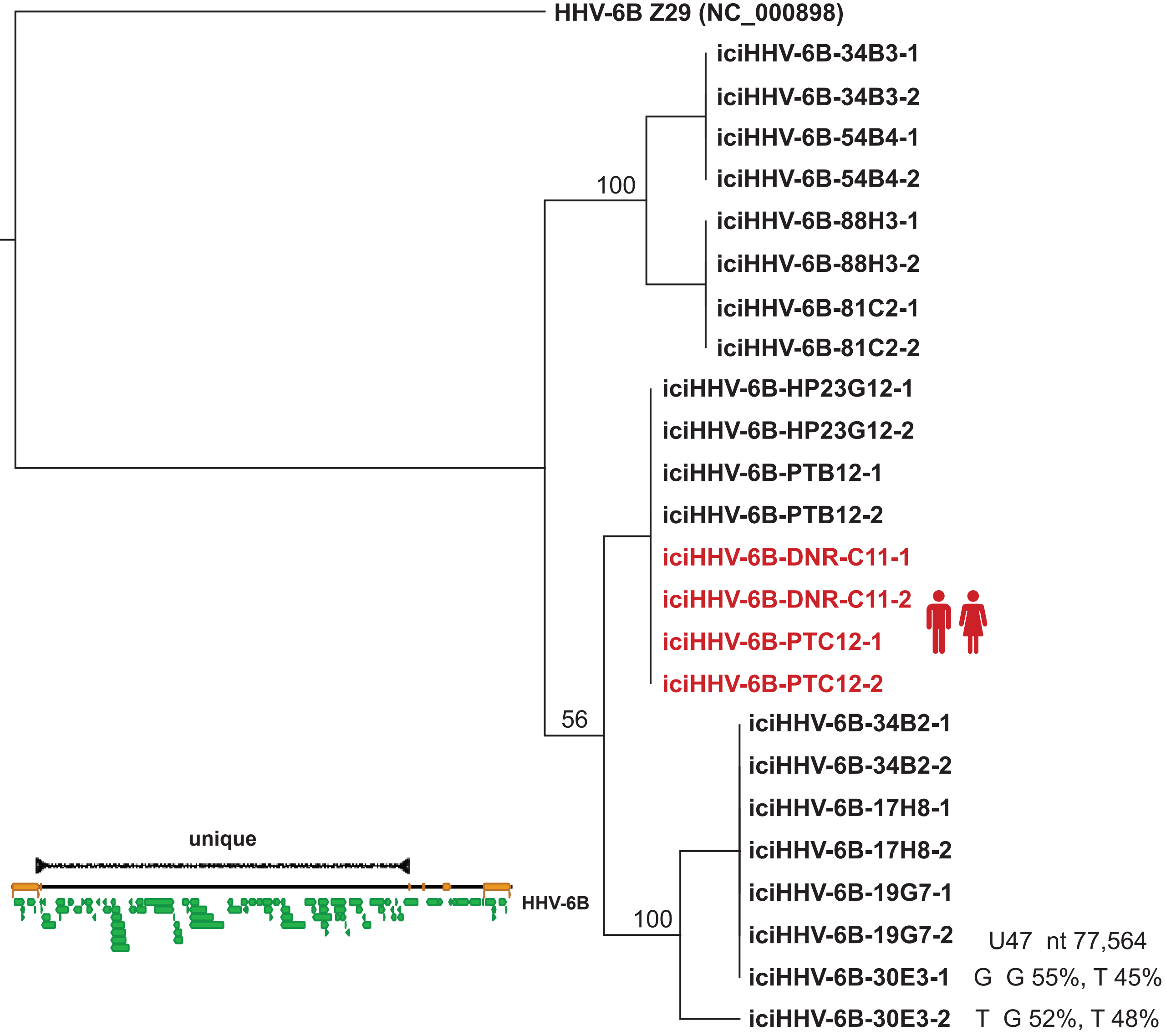
HHV-6B Z29 (NC_000898)
100
iciHHV-6B-34B3-1
iciHHV-6B-34B3-2
iciHHV-6B-54B4-1
iciHHV-6B-54B4-2
iciHHV-6B-88H3-1
iciHHV-6B-88H3-2
iciHHV-6B-81C2-1
iciHHV-6B-81C2-2
iciHHV-6B-HP23G12-1
iciHHV-6B-HP23G12-2
iciHHV-6B-PTB12-1
iciHHV-6B-PTB12-2
iciHHV-6B-DNR-C11-1
iciHHV-6B-DNR-C11-2
iciHHV-6B-PTC12-1
iciHHV-6B-PTC12-2
56
iciHHV-6B-34B2-1
iciHHV-6B-34B2-2
iciHHV-6B-17H8-1
iciHHV-6B-17H8-2
iciHHV-6B-19G7-1
iciHHV-6B-19G7-2
100
iciHHV-6B-30E3-1
iciHHV-6B-30E3-2
unique
HHV-6B
U47 nt 77,564
G G 55%, T 45%
T G 52%, T 48%
