# Supplementary figures and images for: Comparative genomic, transcriptomic, and proteomic reannotation of human herpesvirus 6
Source: BMC Genomics. 2018 Mar 20;19:204. doi: 10.1186/s12864-018-4604-2 (PMC5859498; doi:10.1186/s12864-018-4604-2)

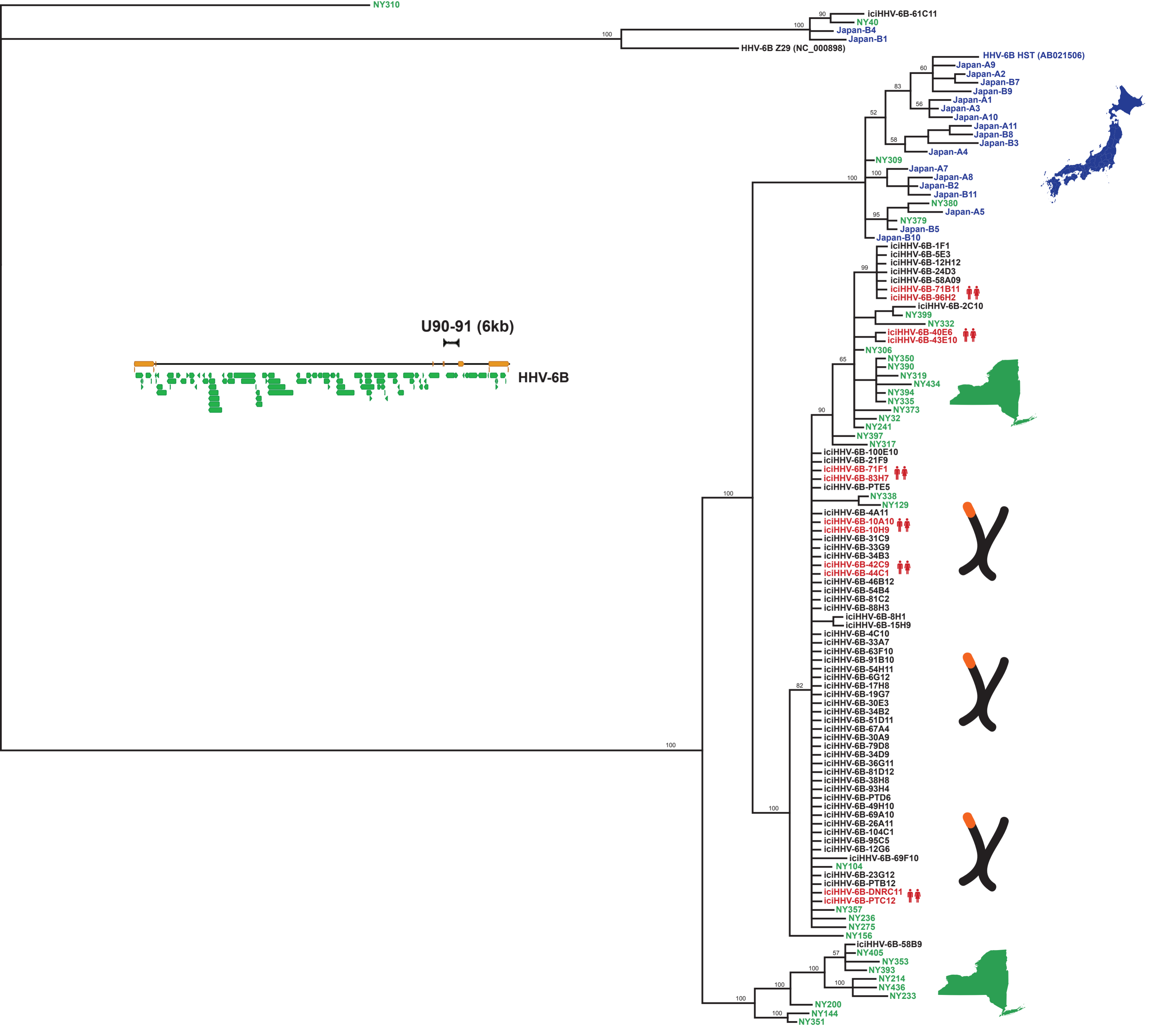

Supplement: Supplementary file 3 — Figure S2. Phylogenetic tree of HHV-6B complete U90/91 and U94/100 loci. HHV-6B genomes were aligned using MAFFT, curated for sequence outside of repeat regions, and phylogenetic trees were constructed using MrBayes along the 6 kb U90/91 (A), and 10 kb U94-100 (B) regions. HHV6-6B NY310 was used as an outgroup. Samples are colored and labeled for origin based on New York (green), Japan (blue), or iciHHV6-B from HSCT recipients or their donors in Seattle (black), as well as whether two genomes were recovered from first-degree relatives (red). Location images purchased from Adobe Stock. (ZIP 656 kb) [file 12864_2018_4604_MOESM3_ESM.zip › FigureS2A-U90phylogeny-v2.pdf]

NY310  
HHV-6B Z29 (NC\_000898)

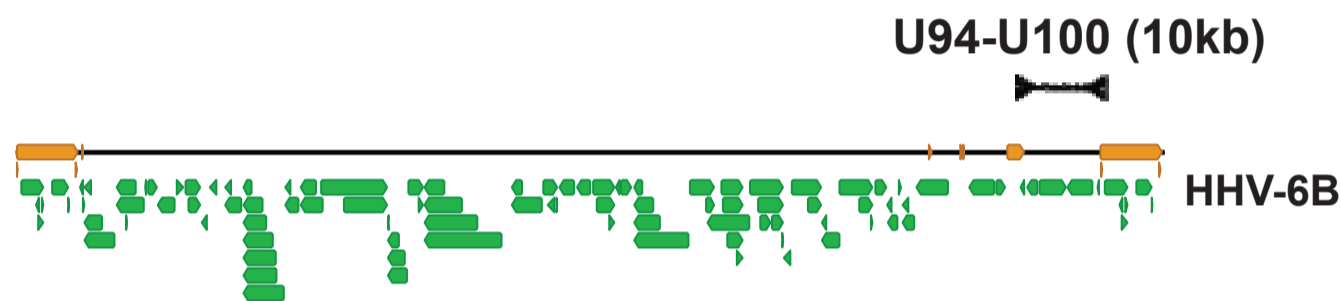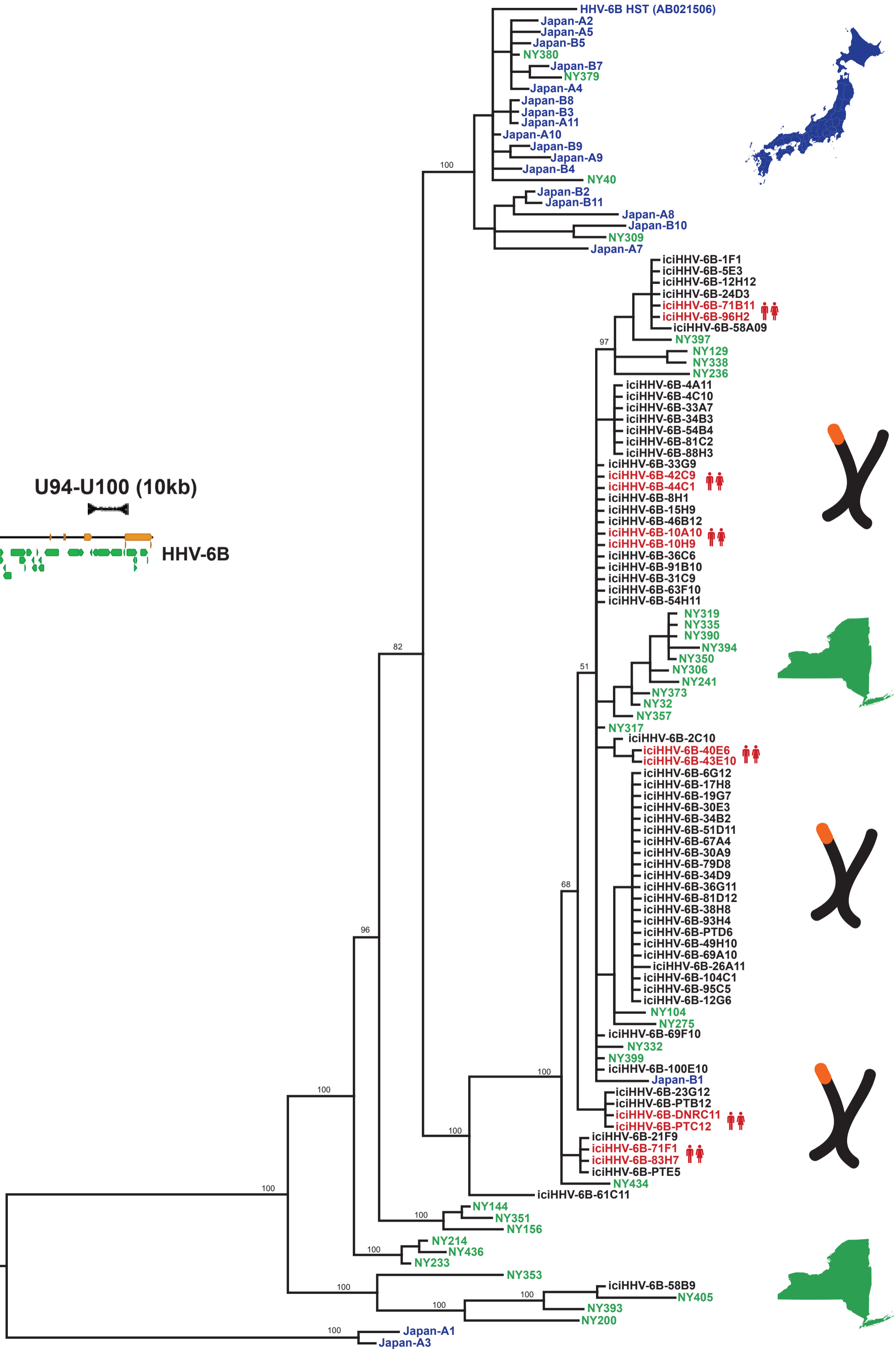

Supplement: Supplementary file 3 — Figure S2. Phylogenetic tree of HHV-6B complete U90/91 and U94/100 loci. HHV-6B genomes were aligned using MAFFT, curated for sequence outside of repeat regions, and phylogenetic trees were constructed using MrBayes along the 6 kb U90/91 (A), and 10 kb U94-100 (B) regions. HHV6-6B NY310 was used as an outgroup. Samples are colored and labeled for origin based on New York (green), Japan (blue), or iciHHV6-B from HSCT recipients or their donors in Seattle (black), as well as whether two genomes were recovered from first-degree relatives (red). Location images purchased from Adobe Stock. (ZIP 656 kb) [file 12864_2018_4604_MOESM3_ESM.zip › FigureS2B-U94-100phylogeny-v2.pdf]
