# Supplementary material for: Comparative genomic, transcriptomic, and proteomic reannotation of human herpesvirus 6
Source: BMC Genomics. 2018 Mar 20;19:204. doi: 10.1186/s12864-018-4604-2 (PMC5859498; doi:10.1186/s12864-018-4604-2)
Supplement: Supplementary file 4 — Figure S3. Non-contiguous gel images of silver stain of HHV-6B Z29 lysates in SupT1 cells or serum-free supernatant run on 10-20% TrisHCl gels in MOPS buffer. (PDF 3011 kb) [file 12864_2018_4604_MOESM4_ESM.pdf]

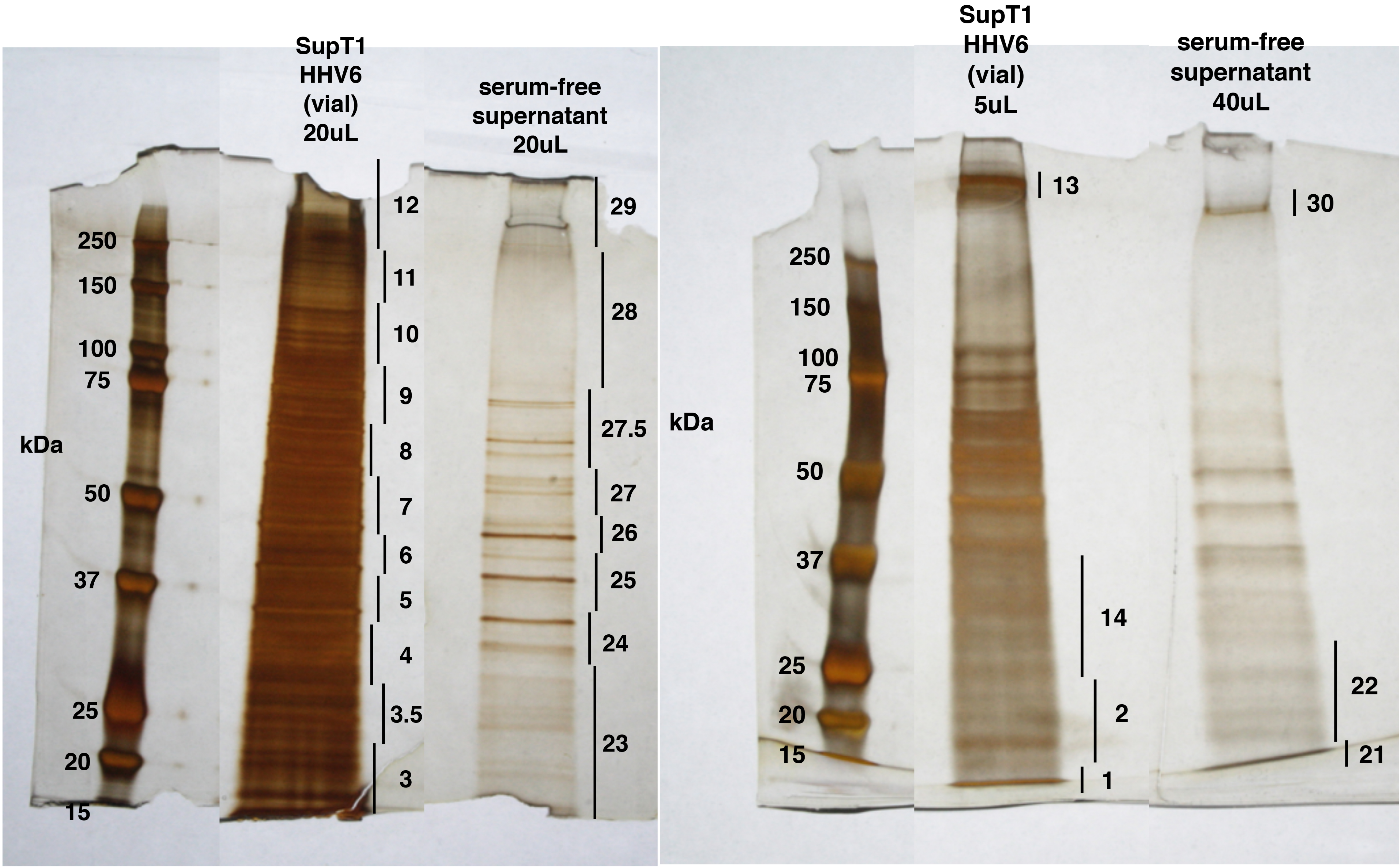

S3 Figure - Non-contiguous gel images of silver stain of HHV-6B Z29 lysates in SupT1 cells or serum-free supernatant run on 10-20% TrisHCl gels in MOPS buffer
