# Supplementary material for: Comparative genomic, transcriptomic, and proteomic reannotation of human herpesvirus 6
Source: BMC Genomics. 2018 Mar 20;19:204. doi: 10.1186/s12864-018-4604-2 (PMC5859498; doi:10.1186/s12864-018-4604-2)
Supplement: Supplementary file 5 — Figure S4. Gel image of silver stain of HHV-6B Z29 lysate in SupT1 cells or serum-free supernatant run on 4-12% TrisHCl gel in MES buffer. (PDF 1335 kb) [file 12864_2018_4604_MOESM5_ESM.pdf]

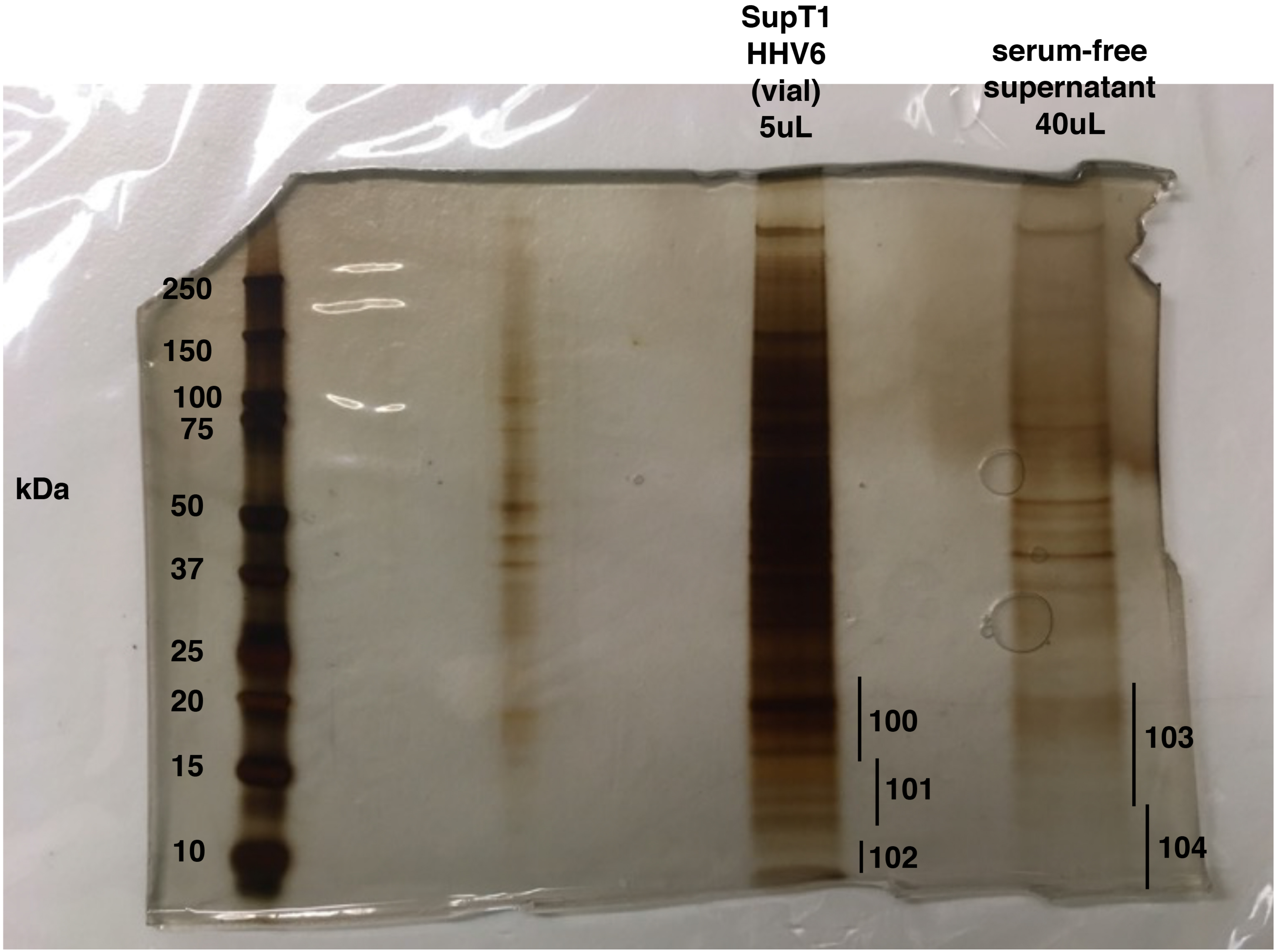

S4 Figure - Gel image of silver stain of HHV-6B Z29 lysate in SupT1 cells or serum-free supernatant run on 4-12% TrisHCl gel in MES buffer
